# Exogenous L-carnitine Administration Ameliorates the Adverse Effects of Heat Stress on Testicular Hemodynamics, Echotexture, and Total Antioxidant Capacity in Rams

**DOI:** 10.3389/fvets.2022.860771

**Published:** 2022-04-06

**Authors:** Hossam R. El-Sherbiny, Amr S. El-Shalofy, Haney Samir

**Affiliations:** Theriogenology Department, Faculty of Veterinary Medicine, Cairo University, Giza, Egypt

**Keywords:** Doppler ultrasonography, heat stress, L-carnitine, nitric oxide, rams, total antioxidant capacity

## Abstract

Heat stress (HS) diminishes the testicular antioxidant defense systems, which adversely affect the testicular blood perfusion. Improving the testicular hemodynamics during HS conditions is of a great impact on the whole reproductive performance in rams. This study aimed to evaluate the ameliorative effects of L-carnitine (LC) on the testicular blood flow and echotextures and also on the total antioxidants (TAC) and nitric oxide (NO) concentrations in the serum during HS conditions in rams. Testicular blood flow was evaluated through scanning of the supra-testicular artery (STA) spectral patterns through pulsed Doppler ultrasonography [peak systolic velocity (PSV), end-diastolic velocity (EDV), time average maximum velocity (TAMAX), resistive index (RI), and pulsatility index (PI)], while the echotexture assessment of testicular parenchyma was performed by a computerized software program. Moreover, TAC and NO concentrations were assayed colorimetrically using the spectrophotometer. There were significant decreases (*P* < 0.05) in values of PSV at 48 and 168 h (23.45 ± 0.39 and 23.37 ± 1.41 cm/s, respectively), and TAMAX at 1, 48, and 168 h (17.65 ± 0.95, 17.5 ± 0.13, and 16.9 ± 1.05 cm/s, respectively) after LC administration compared to just before administration (31.92 ± 1.13 and 21.58 ± 0.92 cm/s, respectively). Values of RI and PI of the examined STA significantly decreased, especially at 1 h for RI (0.45 ± 0.02) and 1 and 48 h for PI (0.66 ± 0.06 and 0.65 ± 0.05, respectively) after LC treatment to 0 h (0.55 ± 0.03 and 0.84 ± 0.06, respectively). The EDV values did not show any significant (*P* < 0.05) changes in all the experimental time points. There were significant (*P* < 0.05) increases in the values of pixel intensity of the testicular parenchyma, especially at 1 and 168 h (78.71 ± 2.50 and 88.56 ± 4.10, respectively) after LC administration, compared to just before administration (69.40 ± 4.75). Serum NO levels tend to increase after LC administration (*P* = 0.07) concerning just before administration. While TAC values showed significant gradual increase and reached the highest values at 168 h (2.75 ± 0.58 mM/l) after LC administration, compared to 0 h (1.12 ± 0.05 mM/l). In conclusion, exogenous LC administration ameliorates testicular hemodynamic disruptions, as measured by spectral Doppler ultrasonography, *via* augmentation of the rams' total antioxidant capacity under HS conditions.

## Introduction

Global warming is one of the most common topics in the world public opinion these days, with many adverse environmental consequences, especially in the field of animal reproduction competence. Environmental thermal stress (TS) triggers oxidative stress (OS) cascade and deteriorates semen quality and fertilizing potential in rams (1, 2), Holstein bulls (3), Shiba goats (4), rabbit bucks (5), and buffalo bulls (6). In rams, elevated testicular temperature induces OS, which in turn diminishes both sperm motility and viability and increases total sperm abnormalities (7). Thermal stress initiates increases in antioxidant defense systems (8) followed by its decline in long-lasting TS *via* decrement of the mRNA expression of the mitochondrial superoxide dismutase (SOD)-1 with a subsequent decrease in SOD protein synthesis and activity, and more generation of superoxide anion (SOA) and cell apoptosis (9).

The positive association between arterial blood flow of the testes and its exocrine (sperm) and endocrine (testosterone hormone) functions has been reported in rams (10, 11), goat bucks (12), donkeys (13), and stud dogs (14). The main route through which testis could be enriched with oxygen and nutrients supply is the testicular artery (TA). Therefore, any alteration in the testicular blood perfusion affects both spermatogenesis and steroidogenesis, with subsequent loss of fertility (15, 16). Spectral Doppler ultrasound is a non-invasive reliable method for an organ function assessment *via* its blood flow measurement (13, 17). The use of testicular blood flow measurement as a potential predictor for male fertility has been applied in many species (14, 18, 19). Resistant index (RI), pulse index (PI), peak systolic velocity (PSV), end-diastolic velocity (EDV), and time-averaged maximum velocity (TAMAX) are the most commonly used parameters for providing a clear judgment on the organ hemodynamics (20).

Digital analysis of the testicular tissue B-mode ultrasound image [testicular echotexture (TE)] has become a useful tool for evaluating the male reproductive functions in different animal species (4, 21). The pixel matrix of the ultrasound image has a score from 0 to 255, with 0 assessed as black color, and 255 corresponds to white color with varying gray color in-between (21). Testicular histology (22), blood flow (4), and sperm-quality parameters (21) governed the degree of TE.

Decreased testicular blood perfusion in heat-stress conditions was reported in rams (10, 11) and goat buck (23). Several attempts have been reported to improve the testicular hemodynamics by administering exogenous treatments in rams and goat bucks with potential outcomes, that finally reflected on the whole male fertility, such as melatonin, gonadotropin-releasing hormone (GnRH), and luteinizing hormone (LH) (12, 19, 24, 25). L-carnitine (LC) is a vitamin-like bioenergetic amino acid that exists ubiquitously in different tissue types, such as heart, skeletal muscle, testes, and epididymis. LC has a substantial role in cell energy production *via* fatty acids (long-chain) transport through mitochondrial membranes and subsequent ATP synthesis *via* the β-oxidation process. Moreover, LC clears the mitochondrial matrix from the accumulated toxic acyl-CoA. L-carnitine protects the cell against OS *via* its antioxidative properties through free radicals (SOA, hydrogen peroxide) capturing and lipid peroxidation inhibition (26–28). Many studies have declared that LC enhances male fertility *via* improving reproductive hormones (29, 30), semen quality (28), antioxidant capacity (28, 31), and gene (GnRH and melatonin) modulation (28, 32, 33). Therefore, the authors postulated that LC administration to heat-stressed rams could alleviate the heat-stress-mediated testicular hemodynamics impairment and improve testicular functions. To the maximum of the authors' knowledge, studying the effect of LC on the rams' testicular hemodynamics in rams housed under heat-stress conditions has not been elucidated. Therefore, this study was aimed, for the first time, to investigate the effects of LC administration on testicular hemodynamics (RI, PI, EDV, PSV, and TAMAX), TE, serum nitric oxide, and total antioxidant concentrations in heat-stressed Ossimi rams.

## Materials and Methods

The experimental procedures of this study were carried out in the summer season (June–August 2021) at the farm of the Theriogenology Department, Faculty of Veterinary Medicine, Cairo University, Giza Governorate, Egypt (30.0276°N, 31.2101°E), following the accreditation of the ethical committee for animal use.

### Animals and Care

Fifteen adult Ossimi rams (55.35 ± 3.5 kg body weight) and aged 3.5 ± 0.16 years, were used in this study. The inclusion criteria were adjusted based on the clinical, cardiovascular, and andrological fitness, based on physical and ultrasound examination. Rams that showed normal heartbeats, pulse rate, capillary refilling time, rectal temperature, and absence of hyperechogenic masses in the testicular ultrasonograms were favored the inclusion criteria. Therefore, only 15 rams were selected, for experimental procedures, out of 23 rams that were submitted to the above-mentioned examinations. Rams' housing was in the open air in a paddock belonging to our department, subjected to normal environmental light, temperature, and humidity. The feeding regime was a mixed formula of concentrate pellets (14% crude protein and energy requirements of 6.39 MJ/kg diet) and green feed with *ad libitum* availability of fresh tape water. They were examined regularly during the experimentation with prophylactic vaccination and deworming programs against endemic diseases in Egypt.

### Rams' Heat-Stress Assessment

The temperature (*T*) and relative humidity (RH) at each time point during the study were over 36°C and 60%, respectively, according to the Egyptian meteorological authority (Cairo, Egypt). The temperature-humidity index (THI) was calculated [THI = *T* – (0.31 – 0.31 RH) (*T* – 14.4)] following a previous study that described the relationship between sheep heat-stress and meteorological conditions (34). Based on the study mentioned later, the rams used in this study were subjected to severe environmental heat stress (THI ≥ 32.5).

### Experimental Protocol

**Figure 1:**
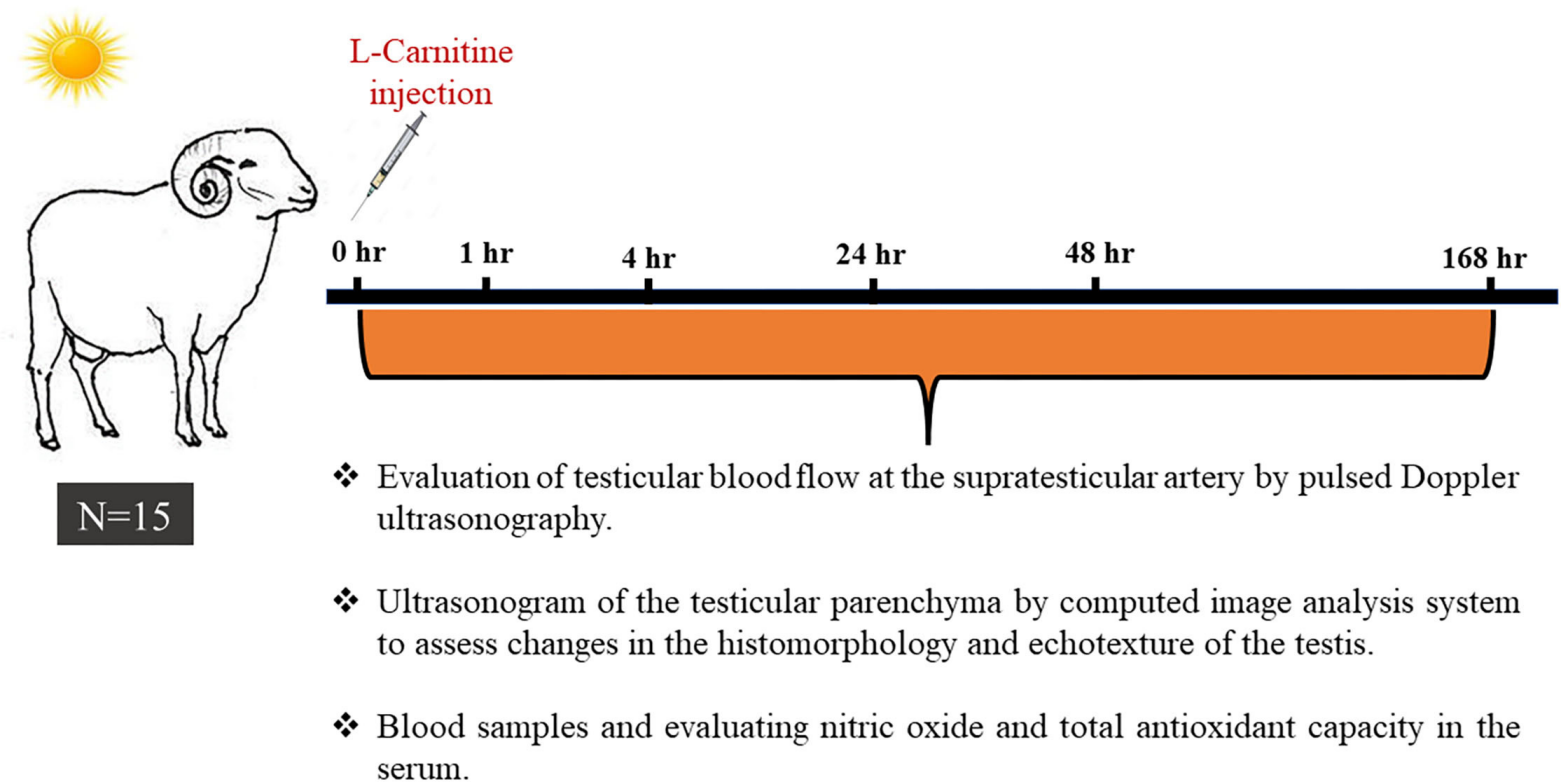
A schematic diagram shows the experimental design, in which fifteen Ossimi rams exposed to environmental heat stress (temperature-humidity index = 32.5) were administered L-carnitine (LC; 20 mg/kg BW; IV) and underwent ultrasound examination and blood sampling just before L-carnitine administration (0 h) and 1, 4, 24, 48, and 168 h post-LC administration.

As explained in Figure 1, heat-stressed rams (*n* = 15) were examined sonographically either by spectral Doppler for the supra-testicular artery of the right and left testis and B-mode scanning of the testicular parenchyma or blood sampling just before (0 h) treatment with LC. Each ram was treated with a single intravenous (jugular vein) injection of LC [20 mg/kg body weight; Mepaco Corporation, Egypt; (35)] and examined at 1, 4, 24, 48, and 168 h after LC administration. The examination time points were selected based on the LC pharmacokinetics, especially the clearance time (36). All the experimental procedures, namely, testicular hemodynamic measurements and echotextures, nitric oxide assessment, and total antioxidant capacity assay, were repeated three times 2 weeks intervals during the same heat-stress conditions.

### Obtaining Blood Samples and Biochemical Analysis

Blood samples (5 ml) were obtained from each ram through jugular venipuncture into plain tubes, just before ultrasonographic assessments at each time point. After centrifugation (15 min) of the drawn blood samples at 1,207 g, the serum samples were harvested and stored (−20°C) till further measurements of nitric oxide metabolites (NOMs) and total antioxidant (TAC) concentrations.

Concentrations of NOMs were measured using commercial kits (nitric oxide kit, Bio-diagnostic, Dokki, Egypt) using a spectrophotometer at 540 nm wavelength. The NOMs inter- and intra-assay coefficient variations were 6.9 and 5.3%, respectively, with assay sensitivity of 0.225 μmol/l in nitrites form (37).

Assessment of TAC was done using commercial kits (total antioxidant capacity, Bio-diagnostic, Dokki, Egypt) spectrophotometrically at a wavelength of 505 nm, based on a previous study (38). The assay rationale is the measurement of the suppressive ability of the sample on sodium benzoate conversion to thiobarbituric acid substances *via* reactive oxygen species (ROS) generated from Fenton's reaction (39).

### Ultrasound Examination

During all the studied time points, sonographic measurements including pulsed-wave Doppler of the right and left supratesticular arteries (STAs) and TE were carried out by the same investigator. An ultrasound device (SonoScape E1V, SonoScape Medical Corp., China) supplied with a 7–14 MHz linear array probe was used for all ultrasonographic measurements. Following a previous study (23), the animals were restrained properly without any sedative agents administration, and the scrotal wool was shaved totally on both sides of each testis till the spermatic cord, and the transducer was loaded with a large amount of ultrasound gel for obtaining clear images for the testes and STA. Just before the testicular inlet, the rams' STA appeared tortuous and convoluted meshed with testicular veins. The instant spectral pattern of the STA (systole and diastole) governed the vessel's discrimination. After a clear view of the STA waveform, pulsed-wave Doppler was used for measurements of the following parameters: (1) PSV (cm/s); (2) EDV (cm/s); and (3) TAMAX (cm/s). From the later-mentioned Doppler parameters, both RI (RI = PSV – EDV/PSV) and PI (PI = PSV – EDV/average velocity) were calculated. All the device settings (brightness, focus, contrast, and gain) were adjusted and fixed throughout the study by the same investigator. The angle between the targeted vessel longitudinal axis and the Doppler beam was ≤60° with 0.5 mm Doppler gate diameter and 50 MHz high-pass filters.

**Figure 2:**
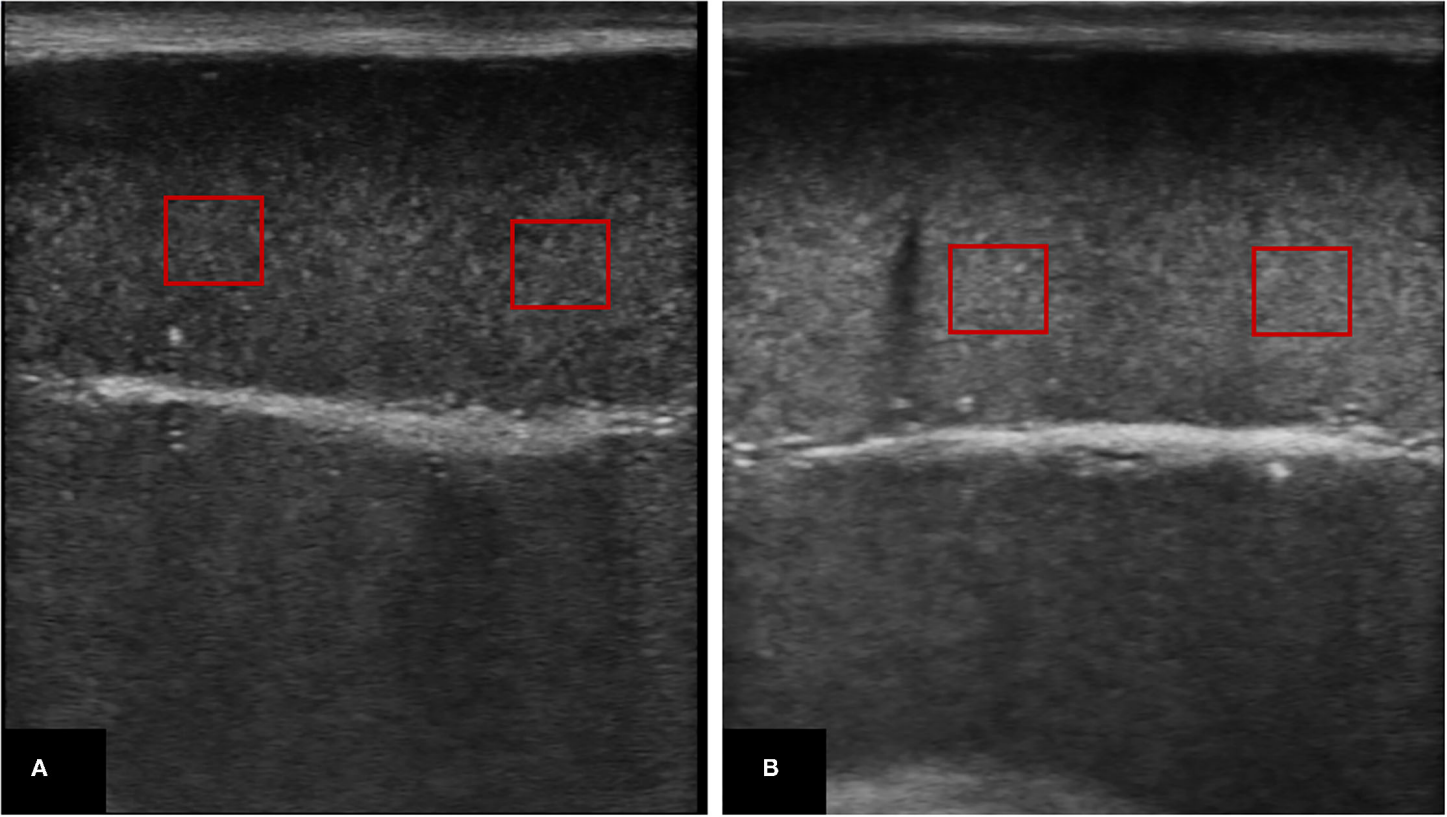
Ultrasonograms of the testicular parenchyma in which echotextures were assessed by placing at least 3 squares (1 cm × 1 cm) and underwent computed digital analysis (Adobe Photoshop 64 CC software); **(A)** represents testicular parenchyma with low echotexture, whereas **(B)** represents higher echotexture.

For TE assessment (22), a clear image, without artifacts, of the longitudinal and transverse aspects of both testes was frozen and saved for further digital computed analysis. The saved images were recalled and examined for testicular echogenicity (TE; the average number of pixels) and pixel heterogeneity (PH; the standard deviation of the pixels) utilizing image analysis software (Adobe Photoshop 64 CC software; Adobe Systems, 2016), by adjusting a 1 cm × 1 cm square in the testicular parenchyma (in 3 different areas) concerning the central positioning of the mediastinum testis (the most hyperechoic part of the testis) (Figure 2).

### Statistical Analysis

The obtained data concerning the testicular hemodynamics (PSV, EDV, RI, PI, and TAMAX), TE and PH, and biochemical analysis (NOMs and TAC) are presented as means ± SEM. The normality and homogeneity of the results were tested using the Kolmogorov–Smirnov test and chi-squared test, respectively. The differences between rams regarding the right and left testes were nonsignificant; therefore, the obtained results were pooled, and the comparisons were done between the different studied time points. Repeated measures ANOVA test was used for discrimination of the differences among the means in the studied time points, and finally with Bonferroni *post hoc* test. GraphPad Prism5 software was used for all the studied statistical assessments. *P* < 0.05 was considered as statistically significant.

## Results

### Effects of LC Administration on Blood Flow Hemodynamic Patterns

The temporal changes in testicular blood flow patterns of the STA of heat-stressed rams administered LC in this study are shown in Table 1. There were significant decreases (*P* < 0.05) in values of PSV at 48 and 168 h (23.45 ± 0.39 and 23.37 ± 1.41 cm/s, respectively) and TAMAX at 1, 48, and 168 h (17.65 ± 0.95, 17.5 ± 0.13, and 16.9 ± 1.05 cm/s, respectively) after LC administration compared to just before administration (31.92 ± 1.13 and 21.58 ± 0.92 cm/s, respectively). Regarding the LC administration effects on RI and PI values of the examined STA, there were significant (*P* < 0.05) decreases, especially at 1 h for RI (0.45 ± 0.02) and at 1 h (0.66 ± 0.06) and 48 h (0.65 ± 0.05) for PI after LC treatment compared to 0 h (RI: 0.55 ± 0.03, PI: 0.84 ± 0.06), whereas, the EDV values did not show any significant (*P* < 0.05) change in all the experimental time points.

**Table 1:** Means ± SEM of the Doppler ultrasound measures of the supra-testicular arteries at the studied time points: just before LC treatment (0 h) and 1, 4, 24, 48, and 168 h post-treatment in Ossimi rams (*n* = 15).

**Parameters**	**0 h**	**1 h**	**4 h**	**24 h**	**48 h**	**168 h**
**PSV (cm/s)**	31.92 ± 1.13	24.95 ± 1.85	35.49 ± 2.61	29.61 ± 2.36	23.45 ± 0.39^*^	23.37 ± 1.41^*^
**EDV (cm/s)**	14.49 ± 0.88	13.80 ± 1.28	16.36 ± 0.74	14.36 ± 1.06	12.23 ± 0.49	11.18 ± 0.84
**RI**	0.55 ± 0.03	0.45 ± 0.02^*^	0.49 ± 0.05	0.47 ± 0.06	0.47 ± 0.03	0.52 ± 0.02
**PI**	0.84 ± 0.06	0.66 ± 0.06^*^	0.77 ± 0.12	0.74 ± 0.12	0.65 ± 0.05^*^	0.73 ± 0.04
**TAMAX (cm/s)**	21.58 ± 0.92	17.65 ± 0.95^*^	23.4 ± 0.59	20.23 ± 0.92	17.5 ± 0.13^*^	16.9 ± 1.05^*^

*PSV, peak systolic velocity; EDV, end-diastolic velocity; RI, resistive index; PI, Pulsatility index; TAMAX, time average maximum velocity; LC, L-carnitine*.

* *Within the same row indicates a significant difference (P < 0.05)*.

### Effects of LC Administration on Testicular Echogenic Properties

The changes in echogenicity of the testicular parenchyma after LC treatment concerning the studied time points in this study are given in Table 2. There were significant (*P* < 0.05) increases in the values of pixel intensity of the testicular parenchyma, especially at 1 and 168 h after LC administration (78.71 ± 2.50 and 88.56 ± 4.10, respectively), compared to just before administration (69.40 ± 4.75). While the PH of the testicular tissue did not exhibit any significant (*P* < 0.05) changes throughout the study time points.

**Table 2:** Means ± SEM of the pixel number and its standard deviation (testicular echogenicity and pixel heterogeneity) of the testicular parenchyma ultrasound B-mode images as analyzed using Photoshop program at the studied time points: just before LC treatment (0 h) and 1, 4, 24, 48 and 168 h post-treatment in Ossimi rams (*n* = 15).

**Parameters**	**0 h**	**1 h**	**4 h**	**24 h**	**48 h**	**168 h**
**TE**	69.40 ± 4.75	78.71 ± 2.50^*^	64.49 ± 2.44	65.42 ± 2.53	69.52 ± 3.91	88.56 ± 4.10^*^
**PH**	10.46 ± 0.48	9.32 ± 0.29	11.5 ± 0.53	11.2 ± 0.48	11.95 ± 0.64	11.34 ± 0.22

*TE, testicular echogenicity; PH, pixel heterogeneity; LC, L-carnitine*.

* *Within the same row indicates a significant difference (P < 0.05)*.

### Effect of LC Administration on Total Antioxidant Capacity and Nitric Oxide Concentrations

The serum total antioxidant capacity and nitric oxide levels in heat-stressed rams administered LC in this work are shown in Table 3. The NO levels in the rams' serum tended to increase (*P* = 0.07) after administration of LC compared to just before administration. Whereas the total antioxidant capacity showed significant (*P* < 0.05) gradual increases and reached the highest values at 168 h (2.75 ± 0.58 mM/l) after LC administration, compared to 0 h (1.12 ± 0.05 mM/l).

**Table 3:** Effects of LC administration on serum NO and TAC concentrations of the heat-stressed rams at 1, 4, 24, 48, and 168 h after treatment vs. 0 h.

**Parameters**	**0 h**	**1 h**	**4 h**	**24 h**	**48 h**	**168 h**	***P*-value**
**NO (umol/L)**	44.93 ± 1.57	53.09 ± 2.33	51.16 ± 3.23	54.94 ± 3.94	49.06 ± 4.99	52.89 ± 4.23	*P* > 0.05
**TAC (mM/L)**	1.12 ± 0.05	1.81 ± 0.22^*^	1.87 ± 0.06^*^	1.64 ± 0.25^*^	1.80 ± 0.13^*^	2.75 ± 0.58^*^	*P* < 0.05

*NO, nitric oxide; TAC, total antioxidant capacity; LC, L-carnitine*.

* *Within the same row indicates a significant difference (P < 0.05)*.

## Discussion

Male reproductive potential under HS conditions faces several obstacles, namely, sperm production decrement, OS, cellular apoptosis, steroidogenesis deficit, testicular hemodynamic disruption, and fertility decline (40). Several trials have been adopted to overcome the negative impact of heat stress in different species by using antioxidants (41–44). L-carnitine, a natural antioxidant, was used extensively in the perspective of cellular bioenergetics, glucose uptake, steroidogenesis, and antiapoptosis under different environmental conditions in different species (26, 29, 30, 35); however and interestingly, this study is the first, according to the best of the authors' update, to use LC for alleviation the adverse effects of heat stress on testicular blood flow (TBF), TE, nitric oxide (NO), and total antioxidants (TAC) concentrations in Ossimi rams. This study showed that treatment of heat-stressed Ossimi rams with LC exerted a significant improvement in testicular blood perfusion. Interpretation of testicular blood flow enhancement is based on RI, PI, PSV, and TAMAX decreases, which explain the elevation of arterial blood perfusion and decrease of the vessel resistance to blood flow. These changes warrant higher testicular blood perfusion (45, 46). The way how LC improved TBF was not certainly clarified in this work; however, many pathways can describe these actions. First, LC may improve TBF through its cardioprotective effect on the myocardium, *via* the promotion of pyruvate oxidation, even at ischemic injury conditions, and helps the heart to recover properly, which affects directly the testicular irrigation with blood (47, 48). Moreover, LC protects the cardiac function through its antiapoptotic properties of the vascular endothelium, evidenced by upregulation of Bcl2 (antiapoptotic) and downregulation of BAX (apoptotic) genes and insulin-like growth factor-1 (49). It would be quite interesting to investigate different parameters of the cardiac and vascular performance regarding the possible testicular blood modulations induced by LC administration in further studies. Healthy vascular endothelial cells produce optimum nitric oxide (potent vasodilator) concentrations resulting in higher blood flow to the reproductive tract (47). Also, LC has a direct effect on NO production in the pulmonary endothelial cells *via* the carnitine acetyltransferase enzyme (50). In addition, LC deficiency induces mitochondrial dysfunction, lessens ATP production and vascular NO synthesis (51). Surprisingly, the differences in NO concentrations between the studied time points in this study were nonsignificant but tended to increase (*P* = 0.07). Earlier studies in men indicated that administration of LC either through oral or parenteral routes provoked NO bioavailability (52, 53). This discrepancy might be due to the differences in the dose (around 1 g/ram), frequency (single dose), and route of administration (IV) of LC treatment in this study compared to the aforementioned reports. The selected dose might be insufficient to elevate the NO concentration, as 1 g of LC may be enough for exerting antioxidant capacity and protecting the cellular components against lipid peroxidation (53), as proposed by the obtained results; whereas, for the elevation of NO, LC should be administered at a dose of at least 3 g daily (52). Second, the role of LC in mitochondrial functions should be considered, through fatty acid transfer, energy production, and removal of excess acyl-CoA, promoting optimum cellular functions in the whole body organs including heart and testes (26, 42, 48). Third, LC as a potent antioxidant protects the testicular cells from the free radicals (SOA and hydroxyl radicals) attacks by capturing them (26), these radicals stimulate the nitric oxide conversion to peroxynitrite (54), thus decreasing its bioavailability which, in turn, lead to decrease TBF. Fourth, LC may affect hypothalamic functions, manifested by elevated blood FSH and LH concentrations which, in turn, modulate testicular hemodynamics and functions (29, 32).

In Ossimi rams, decreased testicular blood flow was reported in the summer season (10). However, there were recent studies indicating a marked increase in testicular blood flow to compensate for increased testicular metabolism under HS conditions (55–57). Indeed, heat stress induces increases in testicular blood perfusion if the heat stress is locally induced on the testes directly (55, 57). However, in our experiment, the rams were exposed to thermal environmental conditions for about 3–4 weeks before the onset of the experiment (summer season). Second, our results may not be consistent with the findings in the above-mentioned literature, as these changes are dependent on whether the testicular temperature exceeded the core body temperature or not. In this study, rams were exposed to ambient temperature (>36°C) and relative humidity (>60%) with temperature-humidity index (32.5) that their effect might be generalized on different body systems. Moreover, the difference in rams' breed adaptability to the thermal conditions may be another factor that should be considered.

Studying the testicular echogenicity provides a complementary noninvasive testicular function evaluation through pixel intensity calculation (21). In this study, there was a significant change in TEs; these changes might be due to increased testicular perfusion with blood. Moreover, the relation between the testicular echogenic and hemodynamic changes has been reported (25) and changes in TE affect the testicular functions (10, 21, 23). Explanation of increases in TE observed at 1 h might be attributed to the higher vascular irrigation. A recent study proposed that higher cellular condensation and vascular irrigation are correlated with higher echogenic changes of the testicular parenchyma of rams, and *vice versa* (58). At 168 h, increases in TE might be attributed to the improvement of total antioxidant capacity compared to 0 h with subsequent amelioration of the OS status that may enhance the functionality of the cellular matrix of the testis including Sertoli, Leydig, and the spermatogenic cells (59).

Heat-stressed rams treated with LC, in this study, experienced significant (*P* < 0.05) higher concentrations of plasma TAC. These findings are in line with many studies conducted in different animal species (26, 53, 60). The improvement in reproductive antioxidant defense systems may be due to the increase in reduced glutathione concentration and activities of glutathione peroxidase, catalase, and SOD enzymes (28, 61, 62), which protect the reproductive organs' defense systems against ROS attacks.

The authors thought that comparing the studied parameters before and after administration of LC is appropriate to test the effects of LC on the same individual of rams (to avoid individual variations). Moreover, to validate the results, the experimental procedures were repeated three times at a 2-week interval in the same conditions. However, the inclusion of the control group in the experimental protocol and examination of a big number of rams may be important to be conducted in a further study. In addition, clarification of the effects of different doses, protocols, routes, and forms of LC on testicular functionality, semen quality, and molecular basics of how LC affects TBF in rams in adverse conditions are valuable to be conducted.

## Conclusion

A single intravenous administration of LC improves testicular hemodynamics (by lessening RI and PI of supra-testicular arteries) and TEs and was followed by an increase in total antioxidants concentrations in Ossimi rams during the heat-stress conditions. However, further studies are needed to be conducted to clarify the effects of different doses, protocols, routes, and forms of LC on testicular functionality, semen quality, and molecular basics of how LC affects TBF in rams in adverse conditions.

## Data Availability Statement

The raw data supporting the conclusions of this article will be made available by the authors, without undue reservation.

## Ethics Statement

The animal study was reviewed and approved by the Ethical Committee for Animal Use, Cairo University, Giza Governorate, Egypt.

## Author Contributions

HE-S: conceptualization, ultrasonographic examination, biochemical analysis, data curation, and manuscript writing. AE-S: conceptualization, experimental design and procedures, and manuscript editing. HS: conceptualization, Doppler examination, biochemical analysis, data validation, statistical analyses, and manuscript reviewing and editing. All authors contributed to the article and approved the submitted version.

## Conflict of Interest

The authors declare that the research was conducted in the absence of any commercial or financial relationships that could be construed as a potential conflict of interest.

## Publisher's Note

All claims expressed in this article are solely those of the authors and do not necessarily represent those of their affiliated organizations, or those of the publisher, the editors and the reviewers. Any product that may be evaluated in this article, or claim that may be made by its manufacturer, is not guaranteed or endorsed by the publisher.

## References

[1] Alves MBR de Andrade AFC de Arruda RP Batissaco L Florez-Rodriguez SA de Oliveira BMM. Recovery of normal testicular temperature after scrotal heat stress in rams assessed by infrared thermography and its effects on seminal characteristics and testosterone blood serum concentration. Theriogenology. (2016) 86:795–805.e2. 10.1016/j.theriogenology.2016.02.034 27045627

[2] Shahat AM Thundathil JC Kastelic JP. Scrotal subcutaneous temperature is increased by scrotal insulation or whole-body heating, but not by scrotal neck insulation; however, all three heat-stress models decrease sperm quality in bulls and rams. J Therm Biol. (2021) 100:103064. 10.1016/j.jtherbio.2021.103064 34503804

[3] Al-Kanaan A König S Brügemann K. Effects of heat stress on semen characteristics of Holstein bulls estimated on a continuous phenotypic and genetic scale. Livest Sci. (2015) 177:15–24. 10.1016/j.livsci.2015.04.003

[4] Samir H Nyametease P Elbadawy M Fathi M Mandour AS Radwan F. Assessment of correlations and concentrations of salivary and plasma steroids, testicular morphometry, and semen quality in different climatic conditions in goats. Theriogenology. (2020) 157:238–44. 10.1016/j.theriogenology.2020.08.002 32818881

[5] Hashem N Abd El-Hady A Hassan O. Effect of vitamin E or propolis supplementation on semen quality, oxidative status and hemato-biochemical changes of rabbit bucks during hot season. Livest Sci. (2013) 157:520–6. 10.1016/j.livsci.2013.09.003

[6] Gonçalves AA Garcia AR Rolim Filho ST da Silva JAR de Melo DN Guimarães TC. Scrotal thermoregulation and sequential sperm abnormalities in buffalo bulls (Bubalus bubalis) under short-term heat stress. J Therm Biol. (2021) 96:102842. 10.1016/j.jtherbio.2021.102842 33627280

[7] dos Santos Hamilton TR de Castro LS de Carvalho Delgado J de Assis PM Siqueira AFP Mendes CM. Induced lipid peroxidation in ram sperm: semen profile, DNA fragmentation and antioxidant status. Reproduction. (2016) 151:379–90. 10.1530/REP-15-0403 26811546

[8] Rizzoto G Kastelic J. A new paradigm regarding testicular thermoregulation in ruminants? Theriogenology. (2020) 147:166–75. 10.1016/j.theriogenology.2019.11.019 31785861

[9] Turrens JF. Mitochondrial formation of reactive oxygen species. J Physiol. (2003) 552:335–44. 10.1113/jphysiol.2003.049478 14561818 PMC2343396

[10] Hedia M El-Belely M Ismail S El-Maaty AMA. Monthly changes in testicular blood flow dynamics and their association with testicular volume, plasma steroid hormones profile and semen characteristics in rams. Theriogenology. (2019) 123:68–73. 10.1016/j.theriogenology.2018.09.032 30292858

[11] Hedia MG El-Belely MS Ismail ST Abo El-Maaty AM. Seasonal variation in testicular blood flow dynamics and their relation to systemic and testicular oxidant/antioxidant biomarkers and androgens in rams. Reprod Domest Anim. (2020) 55:861–9. 10.1111/rda.13696 32374490

[12] Samir H Nyametease P Elbadawy M Nagaoka K Sasaki K Watanabe G. Administration of melatonin improves testicular blood flow, circulating hormones, and semen quality in Shiba goats. Theriogenology. (2020) 146:111–9. 10.1016/j.theriogenology.2020.01.053 32078960

[13] Abdelnaby EA Emam IA Fadl AM. Assessment of the accuracy of testicular dysfunction detection in male donkey (Equus asinus) with the aid of colour-spectral Doppler in relation to plasma testosterone and serum nitric oxide levels. Reprod Domest Anim. (2021) 56:764–74. 10.1111/rda.13916 33595865

[14] Gloria A di Francesco L Marruchella G Robbe D Contri A. Pulse-wave Doppler pulsatility and resistive indexes of the testicular artery increase in canine testis with abnormal spermatogenesis. Theriogenology. (2020) 158:454–60. 10.1016/j.theriogenology.2020.10.015 33049570

[15] Kay G Grobbelaar J Hattingh J. Effect of surgical restriction of growth of the testicular artery on testis size and histology in bulls. Reproduction. (1992) 96:549–53. 10.1530/jrf.0.0960549 1339835

[16] Herwig R Tosun K Pinggera G-M Soelder E Moeller K Pallwein L. Tissue perfusion essential for spermatogenesis and outcome of testicular sperm extraction (TESE) for assisted reproduction. J Assist Reprod Genet. (2004) 21:175–80. 10.1023/B:JARG.0000031251.57848.04 15279325 PMC3455528

[17] EL-Sherbiny H EL-Shahat K Abo El-Maaty A Abdelnaby EA. Ovarian and uterine haemodynamics and their relation to steroid hormonal levels in postpartum Egyptian buffaloes. Bulgarian J Vet Med. (2020). 10.15547/bjvm.2020-0091 [Online ahead of print].

[18] Bollwein H Schulze JJ Miyamoto A Sieme H. Testicular blood flow and plasma concentrations of testosterone and total estrogen in the stallion after the administration of human chorionic gonadotropin. J Reprod Dev. (2008) 54:335–9. 10.1262/jrd.20014 18667792

[19] Samir H Sasaki K Ahmed E Karen A Nagaoka K El Sayed M. Effect of a single injection of gonadotropin-releasing hormone (GnRH) and human chorionic gonadotropin (hCG) on testicular blood flow measured by color doppler ultrasonography in male Shiba goats. J Vet Med Sci. (2015) 14–0633. 10.1292/jvms.14-0633 25715956 PMC4478734

[20] Viana J Arashiro E Siqueira L Ghetti A Areas V Guimarães C. Doppler ultrasonography as a tool for ovarian management. Anim Reprod. (2018) 10:215–22. Available online at: https://www.animal-reproduction.org/article/5b5a6049f7783717068b4694/pdf/animreprod-10-3-215.pdf

[21] Brito L Barth A Wilde R Kastelic J. Testicular ultrasonogram pixel intensity during sexual development and its relationship with semen quality, sperm production, and quantitative testicular histology in beef bulls. Theriogenology. (2012) 78:69–76. 10.1016/j.theriogenology.2012.01.022 22401830

[22] Giffin JL Franks SE Rodriguez-Sosa JR Hahnel A Bartlewski PM. A study of morphological and haemodynamic determinants of TE characteristics in the ram. Exp Biol Med. (2009) 234:794–801. 10.3181/0812-RM-364 19429851

[23] Samir H Nyametease P Nagaoka K Watanabe G. Effect of seasonality on testicular blood flow as determined by color Doppler ultrasonography and hormonal profiles in Shiba goats. Anim Reprod Sci. (2018) 197:185–92. 10.1016/j.anireprosci.2018.08.027 30166078

[24] El-Shalofy AS Hedia MG. Exogenous oxytocin administration improves the testicular blood flow in rams. Andrologia. (2021) 53:e14193. 10.1111/and.14193 34309888

[25] El-Shalofy A Hedia M Kastelic J. Melatonin improves testicular haemodynamics, echotexture and testosterone production in Ossimi rams during the breeding season. Reproduction in Domestic Animals. (2021) 56:1456–63. 10.1111/rda.14010 34459033

[26] Hussein SA Abd El-Hamid OM Hemdan HS. Protective effect of L-carnitine on metabolic disorders, oxidative stress, antioxidant status and inflammation in a rat model of. Int J Biol Chem. (2014) 8:21–36. 10.3923/ijbc.2014.21.36

[27] Li J-L Wang Q-Y Luan H-Y Kang Z-C Wang C-B. Effects of L-carnitine against oxidative stress in human hepatocytes: involvement of peroxisome proliferator-activated receptor alpha. J Biomed Sci. (2012) 19:1–9. 10.1186/1423-0127-19-32 22435679 PMC3338374

[28] Elokil AA Bhuiyan AA Liu H-Z Hussein MN Ahmed HI Azmal SA. The capability of L-carnitine-mediated antioxidant on cock during aging: evidence for the improved semen quality and enhanced testicular expressions of GnRH1, GnRHR, and melatonin receptors MT 1/2. Poult Sci. (2019) 98:4172–81. 10.3382/ps/pez201 31001634

[29] Abdel-Emam RA Ahmed EA. Ameliorative effect of L-carnitine on chronic lead-induced reproductive toxicity in male rats. Vet Med Sci. (2021) 7:1426–35. 10.1002/vms3.473 33724722 PMC8294385

[30] Aziz RLA Abdel-Wahab A El-Ela FIA Hassan NE-HY El-Nahass E-S Ibrahim MA. Dose-dependent ameliorative effects of quercetin and l-Carnitine against atrazine-induced reproductive toxicity in adult male Albino rats. Biomed Pharmacother. (2018) 102:855–64. 10.1016/j.biopha.2018.03.136 29710542

[31] El-Sherbini E-S El-Sayed G El Shotory R Gheith N Abou-Alsoud M Harakeh SM. Ameliorative effects of l-carnitine on rats raised on a diet supplemented with lead acetate. Saudi J Biol Sci. (2017) 24:1410–7. 10.1016/j.sjbs.2016.08.010 28855839 PMC5562480

[32] Genazzani AD Despini G Czyzyk A Podfigurna A Simoncini T Meczekalski B. Modulatory effects of l-carnitine plus l-acetyl-carnitine on neuroendocrine control of hypothalamic functions in functional hypothalamic amenorrhea (FHA). Gynecol Endocrinol. (2017) 33:963–7. 10.1080/09513590.2017.1332587 28573875

[33] Ahmed SD Ahsan S Burney SI. Male fertility: influence of testosterone, luteinizing hormone, and follicle-stimulating hormone on seminal free L-carnitine. Hum Androl. (2013) 3:76–80. 10.1097/01.XHA.0000432480.17007.64

[34] Papanastasiou D Bartzanas T Kittas C editors. Relation between potential sheep heat-stress and meteorological conditions. In: International Conference on Agricultural Engineering AgEng, Zurich. (2014).

[35] Kaçar C Zonturlu AK Karapehlivan M ARI UÇ Ögün M Citil M. The effects of L-carnitine administration on energy metabolism in pregnant Halep (Damascus) goats. Turk J Vet Anim Sci. (2010) 34:163–71. 10.3906/vet-0805-11

[36] Rebouche CJ. Kinetics, pharmacokinetics, and regulation of L-carnitine and acetyl-L-carnitine metabolism. Ann N Y Acad Sci. (2004) 1033:30–41. 10.1196/annals.1320.003 15591001

[37] Abdelnaby EA Emam IA Salem NY Ramadan ES Khattab MS Farghali HA. Uterine hemodynamic patterns, oxidative stress, and chromoendoscopy in mares with endometritis. Theriogenology. (2020) 158:112–20. 10.1016/j.theriogenology.2020.09.012 32956860

[38] Fathi M Salama A El-Shahat K EL-Sherbiny HR Abdelnaby EA. Effect of melatonin supplementation during IVM of dromedary camel oocytes (Camelus dromedarius) on their maturation, fertilization, and developmental rates *in vitro*. Theriogenology. (2021) 172:187–92. 10.1016/j.theriogenology.2021.05.021 34218101

[39] Cortassa S Aon MA Winslow RL O'Rourke B. A mitochondrial oscillator dependent on reactive oxygen species. Biophys J. (2004) 87:2060–73. 10.1529/biophysj.104.041749 15345581 PMC1304608

[40] Nardone A Ronchi B Lacetera N Ranieri MS Bernabucci U. Effects of climate changes on animal production and sustainability of livestock systems. Livest Sci. (2010) 130:57–69. 10.1016/j.livsci.2010.02.011

[41] Sharma S Ramesh K Hyder I Uniyal S Yadav V Panda R. Effect of melatonin administration on thyroid hormones, cortisol and expression profile of heat shock proteins in goats (Capra hircus) exposed to heat stress. Small Rumin Res. (2013) 112:216–23. 10.1016/j.smallrumres.2012.12.008

[42] Ayyat MS Abd El-Latif KM Helal AA Al-Sagheer AA. Interaction of supplementary L-carnitine and dietary energy levels on feed utilization and blood constituents in New Zealand White rabbits reared under summer conditions. Trop Anim Health Prod. (2021) 53:1–8. 10.1007/s11250-021-02723-1 33885998

[43] Shahat A Rizzoto G Kastelic J. Amelioration of heat stress-induced damage to testes and sperm quality. Theriogenology. (2020) 84–96. 10.1016/j.theriogenology.2020.08.034 32947064

[44] Fadl AM Abdelnaby EA El-Sherbiny HR. Supplemental dietary zinc sulfate and folic acid combination improves testicular volume and hemodynamics, testosterone levels and semen quality in rams under heat stress conditions. Reprod Domest Anim. (2022) 00:10. 10.1111/rda.14096 35147249

[45] Ginther O. Ultrasonic Imaging and Animal Reproduction. Cross plains: WI Equiservices Publishing. (1995).

[46] Dickey RP. Doppler ultrasound investigation of uterine and ovarian blood flow in infertility and early pregnancy. Hum Reprod Update. (1997) 3:467–503. 10.1093/humupd/3.5.467 9528912

[47] Stanley WC Lopaschuk GD Hall JL McCormack JG. Regulation of myocardial carbohydrate metabolism under normal and ischaemic conditions: potential for pharmacological interventions. Cardiovasc Res. (1997) 33:243–57. 10.1016/S0008-6363(96)00245-3 9074687

[48] Sharma S Black SM. Carnitine homeostasis, mitochondrial function and cardiovascular disease. Drug Discov Today. (2009) 6:e31–9. 10.1016/j.ddmec.2009.02.001 20648231 PMC2905823

[49] Mohamed MA. Impact of L-carnitine and cinnamon on insulin-like growth factor-1 and inducible nitric oxide synthase gene expression in heart and brain of insulin resistant rats. Am J Biochem Biotechnol. (2010) 6:204–12. 10.3844/ajbbsp.2010.204.212

[50] Sharma S Sun X Agarwal S Rafikov R Dasarathy S Kumar S. Role of carnitine acetyl transferase in regulation of nitric oxide signaling in pulmonary arterial endothelial cells. Int J Mol Sci. (2013) 14:255–72. 10.3390/ijms14010255 23344032 PMC3565262

[51] Sharma S Sud N Wiseman DA Carter AL Kumar S Hou Y. Altered carnitine homeostasis is associated with decreased mitochondrial function and altered nitric oxide signaling in lambs with pulmonary hypertension. Am J Physiol. (2008) 294:L46–56. 10.1152/ajplung.00247.2007 18024721 PMC3970936

[52] Bloomer RJ Tschume LC Smith WA. Glycine propionyl-L-carnitine modulates lipid peroxidation and nitric oxide in human subjects. Int J Vitam Nutr Res. (2009) 79:131–41. 10.1024/0300-9831.79.3.131 20209464

[53] Guzel NA Orer GE Bircan FS Cevher SC. Effects of acute L-carnitine supplementation on nitric oxide production and oxidative stress after exhaustive exercise in young soccer players. J Sports Med Phys Fitness. (2015) 55:9–15. Available online at: https://www.minervamedica.it/en/journals/sports-med-physical-fitness/article.php?cod=R40Y2015N01A0009&acquista=1 25289711

[54] Kissner R Nauser T Bugnon P Lye PG Koppenol WH. Formation and properties of peroxynitrite as studied by laser flash photolysis, high-pressure stopped-flow technique, and pulse radiolysis. Chem Res Toxicol. (1997) 10:1285–92. 10.1021/tx970160x 9403183

[55] Rizzoto G Ferreira J Garcia HM Teixeira-Neto F Bardella L Martins C. Short-term testicular warming under anesthesia causes similar increases in testicular blood flow in Bos taurus vs. Bos indicus bulls, but no apparent hypoxia. Theriogenology. (2020) 145:94–9. 10.1016/j.theriogenology.2020.01.045 32007637

[56] Rizzoto G Hall C Tyberg J Thundathil J Caulkett N Kastelic J. Testicular hyperthermia increases blood flow that maintains aerobic metabolism in rams. Reprod Fertil Dev. (2019) 31:683–8. 10.1071/RD17509 30449297

[57] Adwell CB Brito L Oba E Wilde R Rizzoto G Thundathil J. Arterial blood flow is the main source of testicular heat in bulls and higher ambient temperatures significantly increase testicular blood flow. Theriogenology. (2018) 116:12–6. 10.1016/j.theriogenology.2018.04.022 29758459

[58] Hedia M El-Shalofy A. Ageing affects plasma steroid concentrations and testicular volume, echotexture and haemodynamics in rams. Andrologia. (2022) 54:e14309. 10.1111/and.14309 34755370

[59] Camela ES Nociti RP Santos VJ Macente BI Murawski M Vicente WR. Changes in testicular size, echotexture, and arterial blood flow associated with the attainment of puberty in Dorper rams raised in a subtropical climate. Reprod Domest Anim. (2019) 54:131–7. 10.1111/rda.13213 29989218

[60] Shaker M Houssen M Abo-Hashem E Ibrahim T. Comparison of vitamin E, L-carnitine and melatonin in ameliorating carbon tetrachloride and diabetes induced hepatic oxidative stress. J Physiol Biochem. (2009) 65:225–33. 10.1007/BF03180575 20119817

[61] Roy VK Verma R Krishna A. Carnitine-mediated antioxidant enzyme activity and Bcl2 expression involves peroxisome proliferator-activated receptor-γ coactivator-1α in mouse testis. Reprod Fertil Dev. (2017) 29:1057–63. 10.1071/RD15336 27064025

[62] Cao Y Qu H-j Li P Wang C-b Wang L-x Han Z-w. Single dose administration of L-carnitine improves antioxidant activities in healthy subjects. Tohoku J Exp Med. (2011) 224:209–13. 10.1620/tjem.224.209 21701126

